# Distribution and Frequency of ABO and Rhesus (D) Blood Groups in Somalia: A Retrospective Study on Students of Jazeera University, Mogadishu-Somalia

**DOI:** 10.1155/2022/7981325

**Published:** 2022-01-30

**Authors:** Mohamed Hayir Tahlil Mohamud, Abdullah Dahir H. Aweis, Abdiwahab Sheikh Elmi Adam, Farhia Abdullahi Mohamed, Safia Qasim Fidow, Lul Mohamud Mohamed

**Affiliations:** ^1^Faculty of Medicine and Surgery, Jazeera University, Mogadishu-, Somalia; ^2^Research Unit, Jazeera University, Mogadishu-, Somalia; ^3^Jazeera University Diagnosis and Research Center, Mogadishu-, Somalia; ^4^Faculty of Health Sciences, Jazeera University, Mogadishu-, Somalia; ^5^Pediatric Department, Jazeera University Hospital, Mogadishu, Somalia

## Abstract

**Background:**

There are differences in the distribution and frequency of ABO and D blood groups in different populations of the world. Relatively very little information is available about blood group distributions in the Somali population. Objectives: To identify the distribution and frequency of ABO and D blood groups among the Somali people.

**Methods:**

A retrospective cross-sectional study of 1811 enrolled students of Jazeera University was conducted in Jazeera University diagnosis and research center, Mogadishu-Somalia from December 2017 to December 2020. The result was presented as the frequency of each blood group with percentage. A Fisher's exact test was carried out to test the significant association of the ABO blood group with sex and D antigen with sex.

**Results:**

Blood group O was the most prevalent (61%), followed by A (27%), B (10%), and AB (2%). The D-antigen was present in 97% of participants and 3% were D-negative. The distribution of O+, A+, B+, AB+ among D-positive subjects were 62%, 27%, 9% and 2.0% while that of O-, A-, B- and AB- among D-negative subjects were 57%, 27%, 12% and 3%, respectively. The frequencies of ABO and D-antigens in both male and female subjects were O > A > B > AB. However, this study found no significant difference of ABO with Sex and D-antigens with sex (*P-value*>0.05).

**Conclusions:**

The frequency of ABO and D blood groups among the Somalia population was found to be O > A > B > AB which was similar to those reported from most East African populations.

## 1. Background

Since 1901, more than 20 different blood group systems have been recognized and characterized, but the ABO and Rhesus (D) blood groups remain clinically most important [1]. The ABO blood group which was the first human blood group was discovered by Karl Landsteiner, an American scientist of Austrian origin [2]. Landsteiner discovered that red blood cells carry antigens on their surfaces, and that blood plasma carries antibodies targeted to specific antigens [3]. Later in 1941, the D blood group (D) was defined by both Landsteiner and Wiener [4–6]. Knowledge of the frequencies of blood groups is not only significant to organ transplantation, genetic research, forensic pathology, anthropology, and training ancestral relation of humans, but its prime most important use is for blood transfusion [7]. Since the collapse of the central government in 1991, Somalia lost every public service including health services. Bomb expositions and gunshots are the biggest disasters in the country causing lots of injuries and death tolls with a lack of adequate blood supply for transfusion are the major contributing factors. Apart from that, Somalia includes the top six counties with the highest maternal mortality deaths [8] with post-partum hemorrhage being the major cause [9]. There is a high rate of road traffic injuries, infectious disease, an increasing rate of elective surgeries, and gastrointestinal bleeds [10, 11]. Even though these problems exist, the country has no functional blood bank. When emergency blood donation is required, calling the patient's relatives and friends is the only way to be relied on. Whatever time it takes to get them, the most horrific news to hear is that most of these people do not know their blood groups. Based on current existing emergencies, there is a high need that every individual especially the youth can know his/her blood group for easy blood transfusion. Hence, this study was carried out to determine the distribution of ABO and D blood groups among students of Jazeera University, Mogadishu-Somalia.

## 2. Material and Methods

### 2.1. Subjects

**Figure 1:**
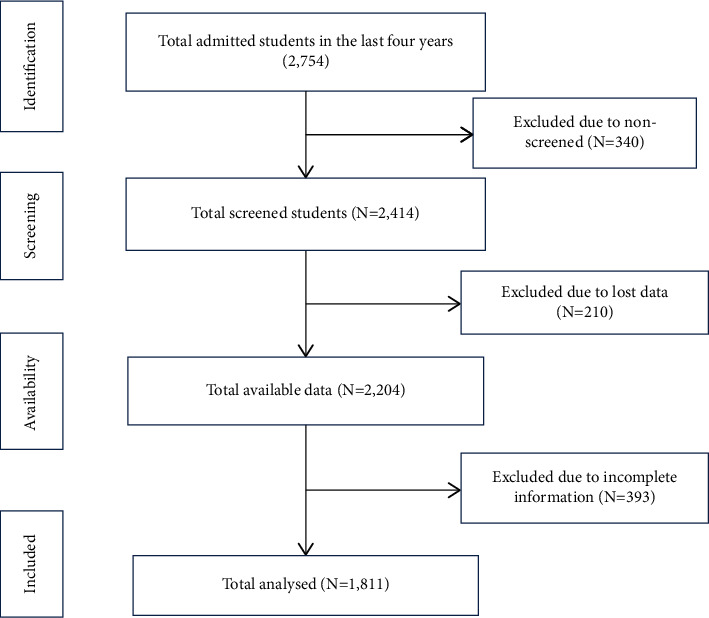
Study flowchart.

The present retrospective study was conducted t in the Jazeera University Diagnosis and Research Centre under Jazeera University Hospital, Mogadishu, Somalia, during the last 4 years from December 2017 to December 2020. Jazeera University diagnosis and research center is an important diagnosis, research and educational center in the center of capital city of Mogadishu. In Jazeera University, every student who enrolls in the University is screened in this center before getting an ID card with his/her name, faculty, department, and blood group. The records of 1811 admitted students in these four years were purposively reviewed as they have complete data available (Figure 1).

A drop of blood from a finger prick was mixed with anti-A, anti-B and anti-D (manufactured by Medsource Ozone Biomedicals Private Limited in India) on separate glass slides and examined for agglutination after one minute incubation.

### 2.2. Statistical Analysis

Data was entered and analyzed using IM-SPSS version 20. The result was calculated as the frequency of each blood group expressed as a percentage. A Fisher's exact test was carried out to test whether the ABO blood group varies with sex and D antigen varies with sex as well. A p-value ≤0.05 was considered to be statistically significant.

## 3. Results

A total of 1811 participants (52% women and 48% men; aged 18-20 years) were analyzed. Blood group O was the most prevalent (61%), followed by A (27%), B (10%) and AB (2%). The D-antigen was present in 97% of participants and 3% were D-negative. The distribution of O+, A+, B+, AB+ among D-positive subjects were 62%, 27%, 9% and 2.0% while that of O-, A-, B- and AB- among D-negative subjects were 57%, 27%, 12% and 3%, respectively. The frequencies of ABO and D-antigens in both male and female subjects were O > A > B > AB. The ABO and D-antigens were no statistically vary with sex (*P-value*>0.05).  Table 1.

## 4. Discussion

This study determined the distribution and frequency of ABO and D antigens among students of Jazeera University, Mogadishu, where there is a lack of data on this subject. The study revealed that the majority type of students' blood group was type O (61%), followed by type A (27%), type B (10%)and the least common was AB (2%). A study conducted in the Somali region of Ethiopia has shown a similar result. This is because the Somali population has been seen to be homogenous and in genetic equilibrium [12]. Studies carried in south Ethiopia [13], Moshi Tanzania [14], Nigeria [15, 16], Uganda [17], and Kenya [18] have all shown that the predominant group to be O, followed by A, B, and the least prevalent to be AB. these trends are consistent with other studies and may indicate that type AB is the least predominant, while type O is the most common on the African continent.

However, there are slight regional variations of ABO blood groups, where in some studies on the continent; as some studies carried out in Western and Central Africa showed that blood group B was predominant to blood group A. For example, studies conducted in Guinea [19], Ghana [20], Nigeria [21], Burkina Faso [22], and Congo [22]. Similar findings were reported from India [23]. These regional diversities may be explained by genetic mapping and the different origins of different races [24].

Several studies in Pakistan [7, 25], Bangladesh [26], North India [26] have shown that blood group B was the predominant type followed by O, A and AB was the least.

Europe can broadly be divided into three areas based on the frequencies of the ABO blood groups. Most of Western Europe shows a high frequency of group A (as compared with the rest of the world) with medium 0 and rather low B. East of the Baltic and Adriatic seas and a line running through Central Germany there is a rise of B, mainly at the expense of 0, and this rise continues steadily to give very high B levels in Central Asia and India. West of the main area of high A, and at several other places on the periphery of Europe, we find very high O levels with B somewhat higher than in the high area. These remarks apply to Iceland, Scotland, and Ireland, too much of Wales and, to a less marked degree, to Northern England [27].

In both males and females, the frequency of ABO blood groups was O > A > B > AB which was similar to the overall ABO blood group distribution among all students. Similarly, in both sexes, the frequencies of D-antigen were also O > A > B > AB in D positive and negative. However, we found that the sex of the subjects was neither associated with the ABO blood group nor with the D-antigen. This was similar to what has been seen in many studies [13, 14, 28, 29].

Determining D antigen in a clinical setting is important to ensure patient safety. In this current study participants who are D negative was only 3% and D positive was 97%. This D positive is consistent with studies conducted in the Somali region of Ethiopia with D positive of 96% [30], in Dhaka, Bangladesh [31] with D positive of 97%, in Moshi, Tanzania with D positive of 98% [14], in Kenya with D positive of 96% [18], in Guinea [32] with D positive of 96%. But slightly higher than studies in South Ethiopia [13] with D positive of 93%, and Burkina Faso [22] with D positive of 92%. However, considering the number of participants in this study, this is not statistically significant. Humans share the same blood groups all over the world, although there are obviously some geographical, regional, and ethnic differences. Ensuring an adequate supply of D negative blood is very important for patient safety.

## 5. Conclusion

Up-to-date knowledge of blood group distribution and frequency under local conditions is essential for the operation of any national health service. To date, there has been a lack of data on this significant topic in Somalia which was under war for the last 30 years with heavy daily expected explosions, gunfire, and high maternal mortality due to bleeding together with a lack of functioning blood banks. This study has found that the frequency of ABO and D blood groups among the Somalia population was O > A > B > AB which was similar to those reported from most East African populations. Similar studies are needed across the country.

## 6. Limitations

This study was conducted among the students of one university in Mogadishu. Hence this may limit the generalizability of the findings to the whole country.

## Figures and Tables

**Table 1:** ABO-D Blood Groups Distribution and Sex.

ABO-D blood groups	Male	Female	Total N (%)
	n (%)	n (%)	
O+	523 (48)	558 (52)	1081 (62)
A+	235 (49)	241 (51)	476 (27)
B+	72 (44)	93 (56)	165 (9)
Ab+	19 (50)	19 (50)	38 (2)
Total	840 (48)	911 (52)	1751 (97)
O-	8 (28)	21 (72)	29 (57)
A-	5 (36)	9 (64)	14 (27)
B-	4 (67)	2 (33)	6 (12)
AB-	2 (100)	0 (0)	2 (4)
Total	19 (37)	32 (62)	51 (3)

(*χ^2^* = 1.395, *P-value* 0.708) for ABO antigen and sex. (*χ^2^* = 1.853, *P-value* 0.796) for D antigen and sex.

## Data Availability

A SPSS and Excel sheet with all primary data is available upon request.
